# Comparative Study of Expected Ecosystem Services and Tree Species Diversity in Residential Plots of Polluted and Unpolluted Neighborhoods in the Copper City of Lubumbashi

**DOI:** 10.1002/pei3.70179

**Published:** 2026-07-14

**Authors:** Jacques Kilela Mwanasomwe, Serge Langunu, Franck Mpetemba wa Kalala, Gilles Colinet, Mylor Ngoy Shutcha

**Affiliations:** ^1^ Ecology, Ecological Restoration and Landscape, Faculty of Agronomic Sciences Université de Lubumbashi Lubumbashi Haut‐Katanga Democratic Republic of the Congo; ^2^ Axis Water‐Soil‐Plant Exchanges, TERRA, Gembloux Agro Bio‐Tech, University of Liège University of Liege Gembloux Namur Belgium; ^3^ Plant Ecology and Biogeochemistry Université Libre de Bruxelles Brussels Belgium

**Keywords:** ecosystem service, neighborhood, phytostabilisation, soil pollution, tree species, urbanization

## Abstract

Natural ecosystem loss and deterioration in the quality of the urban environment due to rapid urbanization associated with soil pollution by mining activity have become a huge dilemma in the mining city of Lubumbashi (DR Congo). This study aimed to assess the importance attributed to trees through the expected services, species choice and their diversity in the polluted and unpolluted neighborhoods of Lubumbashi. Surveys were conducted on 178 residential plots at Penga Penga and Gécamines (polluted neighborhoods) and Kaleja and Kalebuka (unpolluted neighborhoods). In all the neighborhoods, supplying fruits, shade provision and windbreak were identified as the most expected ecosystem services (ES). Penga Penga residents tended to expect more regulating services than Gécamines, Kaleja and Kalebuka residents, who preferred more provisioning services overall. Thirty‐four tree species were identified during the survey, fruiting exotic species dominated by *Mangifera indica* (44.7%) and *Persea americana* (21.2%) were the most frequent in the studied neighborhoods of Lubumbashi, both in the polluted or the unpolluted neighborhoods, but *Acacia auriculiformis* , a non‐fruiting species (4.1%) was only more abundant in the polluted site of Penga Penga. Species richness varied among the neighborhoods, with higher values in unpolluted neighborhoods (Kalebuka and Kaleja: 23 and 22, respectively) than in polluted neighborhoods (Gécamines and Penga Penga: 12 and 18, respectively). As reported in the literature, tree species that cope with polluted soils and provide multiple ES in urban or periurban areas are likely a good option for sustainable phytostabilisation in the Katangan Copperbelt.

## 1 Introduction

The Katangan Copperbelt (KCB), located in the southeastern part of the Democratic Republic of the Congo (DR Congo), is one of the world's most mineral‐rich regions, renowned for its vast reserves of copper and cobalt and for the intensive mining activities that have supported the national economy for decades (François 1973; Cailteux et al. 2005). While mining has significantly contributed to the country's economic development and employment, it has also left behind a serious legacy of environmental degradation. Decades of ore extraction and processing have led to widespread contamination of soils and water bodies by trace metals such as copper (Cu), cobalt (Co), and lead (Pb) (Katemo Manda et al. 2010; Narendrula et al. 2012; Shutcha et al. 2010, 2015). These pollutants pose critical risks not only to terrestrial and aquatic ecosystems but also to public health and urban livelihoods, especially in areas where former or active mining sites are now encroached upon by expanding urban settlements. In cities like Lubumbashi, the capital of Haut‐Katanga Province, entire neighborhoods have been created on or near contaminated lands, exposing communities to prolonged and diffuse sources of contamination (Banza et al. 2009; Useni et al. 2018; Mwanasomwe 2022). In this context, restoring ecosystem functions and services in trace metal (TM) contaminated sites has become a key challenge for sustainable urban development (Potschin and Haines‐Young 2011; Cundy et al. 2016). Among the range of remediation strategies, urban tree planting has emerged as a particularly promising approach, known as phytostabilisation with tree species. Trees help stabilize contaminated soils, reduce erosion, and limit the mobility of trace metals, thereby mitigating environmental and health hazards (Pilon‐Smits 2005; Cundy et al. 2016). Beyond their remediation role, trees provide essential ecosystem services such as carbon sequestration, air purification, microclimate regulation, and habitat for local biodiversity. They also enhance the visual and psychological quality of urban spaces, which is crucial in densely populated and socio‐economically vulnerable areas like the Katangan Copperbelt (McPherson 1992; Nowak and McPherson 1993; Killicoat et al. 2002; Lohr et al. 2004). The creation of vegetated land in TM‐polluted areas led to the improvement of various ecological functions and ecosystem services in TM‐contaminated areas of the KCB. The studies conducted in TM contaminated sites of the KCB showed that the vegetated contaminated soils in comparison to bare soils reduced soil erosion and metal dispersion (Shutcha et al. 2015; Mwanasomwe, Langunu, Shutcha, and Colinet 2022), increased the diversity of soil macrofauna and fertility (Mwanasomwe, Langunu, Nkulu, et al. 2022; Mashagiro, Mujinya Bazirake, et al. 2024; Mashagiro, Mujinya, et al. 2024), and trees can produce wood for charcoal with reduced metal content and edible fruits if safe weekly consumption is respected (Mwanasomwe, Langunu, Shutcha, and Colinet 2022; Langunu et al. 2023). However, for tree planting initiatives to yield lasting ecological and social benefits (Shackleton et al. 2015), the selection of appropriate species is paramount. Not all species can survive or thrive in metal‐contaminated soils. Moreover, trees must also fulfill the practical and cultural expectations of local communities. In many urban and peri‐urban neighborhoods, residents value trees not only for their environmental functions but also for their ability to provide edible fruits, medicinal products, fuelwood, or building materials. This underscores the need for integrated approaches that align ecological suitability with local needs and preferences (Mwanasomwe 2022). Over the past two decades, the polluted areas around Lubumbashi have become increasingly inhabited, and a growing variety of tree species is being observed in residential plots (Mwanasomwe 2022). This observation raises important questions about the role of spontaneous or deliberate tree planting in polluted urban contexts. Therefore, this study aimed (1) to assess residents' expectations regarding the ecosystem services (ES) provided by trees in their residential plots, and (2) to analyze species abundance and distribution in both trace metal‐polluted and unpolluted neighborhoods of Lubumbashi. This research is part of a broader effort to evaluate the potential of tree planting as a strategy for restoring degraded urban ecosystems in the Katangan Copperbelt. It seeks to determine whether the tree species currently established in residential areas are ecologically viable in contaminated soils and whether they are expected to deliver socio‐economic and environmental benefits that resonate with the needs of local communities. Ultimately, this study highlights the importance of integrating social perceptions into ecological restoration planning and demonstrates the value of participatory, site‐specific approaches in designing sustainable solutions for complex environmental challenges in mining‐impacted regions.

## 2 Materials and Methods

### 2.1 Study Area

The survey was conducted in Lubumbashi, one of the biggest cities in the Katangan Copperbelt (KCB), DR Congo, with large areas polluted by mining activities of the Gécamines copper plant since the beginning of the 20th century. Four different neighborhoods were considered for this study: Penga Penga (a newly inhabited area) and Gécamines (a long‐time inhabited area), which are both polluted in trace metals of the metalliferous fallout from the source Gécamines copper plant, the source of contamination (Mpundu 2010) located at a distance of 0 to 2 km from the source of pollution, and at Kalebuka (a newly inhabited area) and Kaleja (a long‐time inhabited area), the unpolluted neighborhoods located at 6 to 7 km from the source of contamination. The survey was conducted on 178 participants (78 at Penga Penga, 56 at Kalebuka and 22 at Gécamines and Kaleja). In a long‐time inhabited neighborhood, the number of participants was so limited, as in most cases the people who owned the plots and planted trees were no longer present (because he/she died, moved or put renters). The mean area of residential plots was Gécamines: 310 m^2^, Penga Penga: 673 m^2^, Kalebuka: 688 m^2^ and Kaleja: 952 m^2^.

### 2.2 Survey Design

The sampling in the neighborhoods was made following the residential plots. Since the number of plots was not known beforehand, the surveyed plots were chosen by randomly selecting a street in the studied neighborhood, then designating the third street by jumping two streets. After the first selected plot in a street for survey, the next residential plot was systematically selected by counting five plots on the right side and five on the left and so on. The respondent was either the one who planted the tree or the one who participated in the tree planting activity at the residential plots. In each surveyed plot, trees were assessed by their number and species.

### 2.3 Soil Sampling

Ten composite soil samples were collected in each of the four studied neighborhoods (Penga Penga, Gécamines, Kalebuka, and Kaleja) for a total of 40 samples (Table 1). The total organic carbon, pH, potassium, magnesium, and calcium were analyzed at the laboratory of the Provincial Centre for Agriculture and Rurality (CPAR) of La Hulpe, Belgium, and copper, cobalt, zinc, and lead at the laboratory of Gembloux Agro‐Bio Tech, Axe Echanges Eau‐Sol Plant, University of Liège.

**TABLE 1:** Chemical characteristics of soil collected inside residences from four neighborhoods in Lubumbashi (Penga Penga, Gécamines, Kalebuka, Kaleja).

Chemical properties	Penga Penga	Gécamines	Kalebuka	Kaleja	Reference^a^
pH_water_	6.9 (5.1–7.7)	—	7.5 (5.9–8.3)	—	5.5 (4.9–6.8)
pH_KCl_	6.7 (4.9–7.9)	—	6.8 (5.2–8.2)	—	4.1 (3.8–5.8)
TOC (%)	1.4 (0.6–3.3)	—	1.2 (0.4–2.0)	—	2.3 (1–5)
P (mg 100 g^−1^)	9.2 (0.4–29.8)	—	4.8 (0.7–9.0)	—	—
K (mg 100 g^−1^)	22.4 (7.9–50.9)	—	28.6 (27.5–29.6)	—	—
Ca (mg 100 g^−1^)	198 (26–638)	—	498 (100–896)	—	—
Mg (mg 100 g^−1^)	13.3 (3.4–32.1)	—	19.8 (12.6–26.9)	—	—
Cu (mg kg^−1^)	1643 (893–6301)	6140 (644–23,362)	344.5 (162–859)	149 (59–375)	191 (20–456)
Co (mg kg^−1^)	98.5 (38–217)	322 (59–1519)	24.5 (21–96)	12 (6.3–56)	20 (7.1–38)
Zn (mg kg^−1^)	471 (224–1041)	1742 (645–6290)	174.5 (109–286)	98 (34–196)	69 (26–180)
Pb (mg kg^−1^)	228 (63–1103)	277 (62–1033)	46 (29–67)	15 (7.3–33)	100 (7.0–82.3)

*Note:* Average (minimum‐maximum), *n* = 10, macronutrients extracted with EDTA and pseudo‐total TM extracted with aqua regia.

^a^ Shutcha et al. (2018).

### 2.4 Resident Characteristics

The majority of Penga Penga, Kalebuka and Kaleja residents occupied their plots from 2000 to 2009, while most of Gécamines' residents (55%) occupied earlier (from 1980 to 1989). The proportion of men was slightly lower than that of women at Penga Penga (37.5% male and 62.4% female), but slightly higher at Kalebuka and Kaleja (45.5% male and 54.5% female). Lastly, the prevailing educational level of residents was primary in all neighborhoods, and most respondents were aged between 37 and 56 years.

### 2.5 Functional Traits of Tree Species and Potential Ecosystem Services

Tree species present a high variability of characteristics that contribute to human well‐being. These characteristics, taken as ecosystem services in ecology, are to be considered in tree choice and purpose for the sustainable phytostabilisation of areas. Table 2 outlines some reported ES documented from the most abundant species found in residential plots of the four Lubumbashi neighborhoods.

**TABLE 2:** Reported ecosystem services documented from the most abundant species found in residential areas of four Lubumbashi neighborhoods.

Scientific name	Ecosystem services	Utilization	Dyservice	Country or region	References
*Mangifera indica*	Provisioning	Food (fruits), medicinal (leaves, barks), fuel wood (tree branches), cosmetic and edible oil (kernel)	Unreported	DRC, India	Pradhan et al. (2020), Nadeem et al. (2016), Shah et al. (2010)
	Regulating and supporting	Shade			
	Cultural	Maintaining social cohesion			
*Persea americana*	Provisioning	Food (fruits), medicinal (leaves)	Unreported	Cameroun, Tanzania, India, Nigeria	Wagner et al. (2019), Temgoua et al. (2018), Yasir et al. (2010), Adeyemi et al. (2002)
	Regulating and supporting	Shade			
*Carica papaya*	Provisioning	Food (fruits), medicinal (leaves and seeds), latex, soap (leaves)	Unreported	DRC, Nepal	Silva et al. (2007), Makumbelo et al. (2002)
*Psidium guajava*	Provisioning	Food (fruits), medicinal (leaves), tool handle (tree branches)	Invasive	Kenya, DRC, Vietnam	Nguyen et al. (2020), Kawawa et al. (2016)
*Acacia auriculiformis*	Provisioning	Fuel wood (charcoal and firewood)	Invasive, depleting groundwater	RDC, Malaysia	USAID (2019), Asif et al. (2017), Proces et al. (2018), Boldrini et al. (2017), Bisiaux et al. (2009), Useni et al. (2022, 2024)
	Regulating and supporting	N‐fixing, Reclamation			
*Citrus limon*	Provisioning	Food (fruits), medicinal (fruits and leaves), cosmetic (fruits)	Unreported	India, Jordan	Klimek‐Szczykutowicz and Ekiert (2020), Al‐Qudah et al. (2018), Pal (2017)
*Syzygium guineense*	Provisioning	Food (fruits), fuel wood, medicinal (leaves)	Unreported	DRC, Benin	Badou et al. (2019), Boldrini et al. (2017)
*Brachystegia spiciformis*	Regulating and supporting	Shade or shelter	Unreported	East and southern Africa	Degreef et al. (2020), Mgumia (2017)
	Provisioning	Food (ectomycorrhizal mushrooms), Fuel wood, timber, fodder, medicinal			
	Regulating and supporting	Shade or shelter			
*Eucalyptus spp*	Provisioning	Timber, furniture, paper, fuel wood, Medicinal and natural pesticide (leaves)	Soil acidification, depleting groundwater and nutrients, threat for biodiversity	Ethiopia, India	Zegeye (2010), Batish et al. (2008); Sangha and Jalota (2005); Lemenih and Bekele (2004); Pohjonen and Pukkala (1990)
	Regulating and supporting	Shade or shelter			
*Pinus sylvestris*	Provisioning	Timber, furniture, paper	Soil acidification	Scandinavia, Canada	Durrant et al. (2016); Brand et al. (1986)
	Regulating and supporting	Reclamation			
*Annona muricata*	Provisioning	Food, medicinal, insecticide	Poison (leaves)	Benin, Mexico	Gbonsou et al. (2020), Gavamukulya et al. (2017), Le Donne et al. (2017)
*Leucaena leucocephala*	Provisioning	Timber, fuelwood, forage	Invasive	Tropical regions	de Sousa Machado et al. (2020), Richardson and Rejmánek (2011), Whitesell and Parrotta (2008)

### 2.6 Data Analysis

Chi‐squared test was used to treat categorical responses on the expected ES from residents in neighborhoods through the contingency table and at a p 0.05 comparison. The relative abundance of trees was calculated from the total number of tree individuals per species in all four neighborhoods surveyed. The comparison of the recorded tree numbers per residential plots in the four neighborhoods was performed using the non‐parametric test of Kruskal–Wallis, as data were not normally distributed after the Shapiro–Wilk test (*p* < 0.05). All the statistical analyses were performed using R Studio software.

## 3 Results

### 3.1 Expected Ecosystem Services in the Residential Plots of Urban Areas

Table 3 shows the expected ES by the residents from tree planting at Penga Penga, Gécamines, Kalebuka and Kaleja neighborhoods. The provisioning ES cited were fruits and medicinal resources supplied by trees. The regulating and supporting ES were the windbreak effect, cooling air locally, shade provision, dust decrease in air, and prevention of erosion. The cultural ES that were expected by residents was related to aesthetics.

**TABLE 3:** Expected ecosystem services by residents from tree planting in four neighborhoods of Lubumbashi.

Category of the ecosystem services	Specification	Penga Penga	Gécamines	Kalebuka	Kaleja	All (178)	χ^2^ (0.05)
**Provisioning**							
Food	Supplying edible fruits	50.0% (39)	90.9% (20)	78.6% (44)	90.9% (20)	69.1% (123)	25.5***
Medicinal resources	Supplying medicinal plants	2.6% (2)	13.6% (3)	5.4% (3)	18.2% (4)	6.7% (12)	8.6*
**Regulating and supporting**							
Moderation of extreme events	Windbreak	53.9% (42)	22.7% (5)	53.6% (30)	59.1% (13)	50.6% (90)	7.9*
Local climate and air quality regulation	Cooling the air locally	5.1% (4)	18.2% (4)	8.9% (5)	18.2% (4)	9.6% (17)	5.6NS
Local climate and air quality regulation	Shade provision	61.5% (48)	59.1% (13)	42.8% (24)	72.7% (16)	56.7% (101)	7.5NS
Local climate and air quality regulation	Decreasing dust in the air	7.7% (6)	4.6% (1)	0.0% (0)	0.0% (0)	3.9% (7)	6.1NS
Erosion prevention and maintenance of soil fertility	Preventing soil erosion due to rainfall and wind	1.3% (1)	0.0% (0)	0.0% (0)	0.0% (0)	0.6% (1)	1.3NS
**Cultural services**							
Aesthetic appreciation and inspiration for culture	Aesthetic	3.9% (3)	0.0% (0)	1.8% (1)	9.1% (2)	3.4% (6)	3.5NS

*Note:* Number of residents surveyed in the neighborhoods: Penga Penga (78 residents) and Gécamines (22 residents): Trace metal‐polluted neighborhoods; Kalebuka (56 residents) and Kaleja (22 residents) unpolluted neighborhoods.

In all the neighborhoods supplying fruits, shade provision, and windbreak were identified as the most expected ES, followed by cooling the air locally and supplying medicinal plant services, and lastly improving air quality by dust decrease was only cited in the polluted neighborhoods of Penga Penga and Gécamines. Aesthetic service was cited at Kaleja, Penga Penga, and Gécamines, and the service of preventing soil erosion due to rainfall and wind was only mentioned one time at Penga Penga (Table 3). Fruit supply by trees was the most expected ES (61.1%), followed by shade provision (56.7%) and windbreak effect (50.6%). The other ES had a frequency of citation varying from 23.9% to 0.6%. There were differences in the frequency of the expectations on supplying edible fruits and medicinal plants and on moderation of extreme events (wind break), while no significant difference was shown concerning other services in all the neighborhoods after a Chi‐squared test. More expectations were noticed for supplying edible fruits at Kaleja and Gécamines (90.9%) and Kalebuka (78.6%) than at Penga Penga (50.0%), where residents expect to benefit from provisioning services in the same way as the regulating service. The expectation of gaining windbreak service was lower at Gécamines (22.7%) compared to the other 3 neighborhoods.

### 3.2 Tree Species Planted in the Four Neighborhoods of Lubumbashi

**FIGURE 1:**
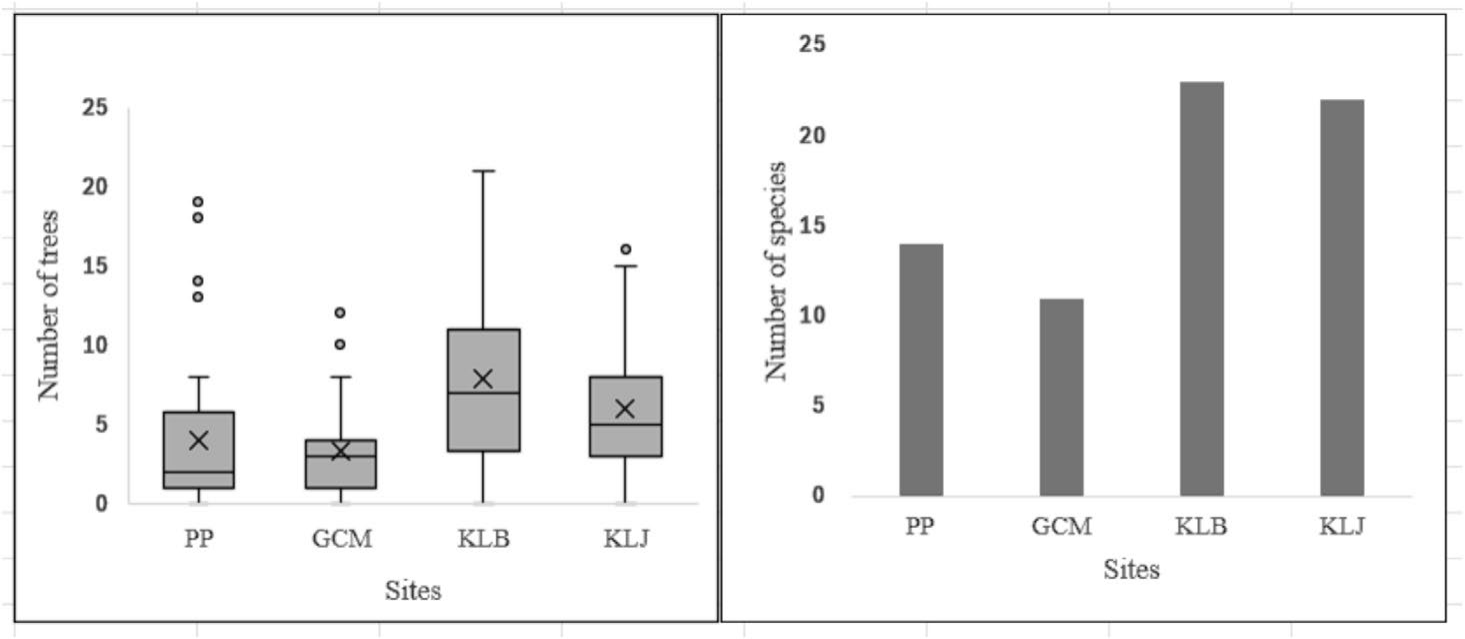
Descriptive statistics of the number of trees per residential plot, and species richness in four neighborhoods. (GCM, Gécamines; PP, Penga Penga; KLB, Kebuka; KLJ, Kaleja), *n* = 22 residential plots (The mean area of plots = 656.3 m^2^). The non‐parametric test of Kruskal–Wallis at *p* < 0.05 was applied; the whiskers represent the minimum and the maximum numbers of trees in the plots, and the values from the first quartile to the third quartile represent the range distribution of most of the data. The bar‐plots represent the total number of species per site.

The survey counted 486 trees in all 88 plots (22 plots for each neighborhood), with Kalebuka contributing with a higher number (181 trees, 33.7%), followed by Kaleja (136 trees, 25.3%) while lower contributions were found at Penga Penga (92 trees, 17.1%) and Gécamines (77 trees, 14.3%). The number of trees per residential plot varied following the total number of trees found in the overall neighborhoods. The residences located in the polluted neighborhoods with metal‐rich soil have a lower number of trees per residence (Penga Penga: 4.8, 0–18; Gécamines: 3.7, 0–12) compared to those with the non‐metal‐rich soil (Kalebuka: 9.2, 3–21; Kaleja: 6.7, 1–15) (Figure 1). The non‐parametric test of Kruskal‐Wallis showed the statistical difference (*p* < 0.05) in the number of trees per residential plot between the unpolluted neighborhoods (Kalebuka and Kaleja) and the polluted neighborhoods (Penga Penga and Gécamines). Thirty‐four tree species were identified during the survey (Table 4). Among them, the fruiting exotic species were the most abundant either in the metal‐polluted or unpolluted neighborhoods. *M. indica* was the most abundant species with a total of 217 individuals i.e., 44.7% of the total number of trees identified, followed by *P. americana* (21.2%). The other fruit species had a frequency lower than 10%. The frequencies of all the native miombo species were lower than 3%.

**TABLE 4:** Tree species identified in four neighborhoods of Lubumbashi.

Especes	PP	GCM	Kalebuka	Kaleja	Total	% of total	Status of species
*Mangifera indica*	27	41	85	64	217	40.9	Exotic
*Persea americana*	22	19	45	17	103	19.4	Exotic
*Elaeis guineensis*	6	4	13	9	32	6.0	Exotic
*Carica papaya*	2	2	12	12	28	5.3	Exotic
*Psidium guajava*	6	4	6	10	26	4.9	Exotic
*Acacia auriculiformis*	17	0	1	2	20	3.8	Exotic
*Citrus lemon*	2	1	11	3	17	3.2	Exotic
*Musa sinensis*	4	1	9	2	16	3.0	Exotic
*Syzygium guineense*	3	2	3	3	11	2.1	Native
*Brachystegia spiciformis*	7	0	1	0	8	1.5	Native
*Eucalyptus* sp	0	5	1	1	7	1.3	Exotic
*Pinus sylvestris*	0	0	2	4	6	1.1	Exotic
*Anona muricata*	0	2	0	3	5	0.9	Exotic
*Leucaena leucocephala*	2	1	0	2	5	0.9	Exotic
*Combretum molle*	0	0	2	2	4	0.8	Native
*Phyllanthus muellerianus*	1	0	2	1	4	0.8	Native
*Moringa oleifera*	0	0	0	3	3	0.6	Exotic
*Morus rubra*	0	0	1	2	3	0.6	Exotic
*Albizia adiantifolia*	0	0	1	0	1	0.2	Native
*Albizia lebbeck*	1	0	0	0	1	0.2	Exotic
*Baphia bequaertii*	0	0	1	0	1	0.2	Native
*Citrus sinensis*	0	0	1	0	1	0.2	Exotic
*Cocos nucifera*	0	0	0	1	1	0.2	Exotic
*Cupressus lusitanica*	0	0	0	1	1	0.2	Exotic
*Dalbergia boehmii*	0	0	0	1	1	0.2	Native
*Delonix regia*	1	0	0	0	1	0.2	Exotic
*Eucalyptus cameldulensis*	0	0	1	0	1	0.2	Exotic
*Julbernadia paniculata*	0	0	1	0	1	0.2	Native
*Ochna scheinfurthiana*	0	0	0	1	1	0.2	Native
*Pericopsis angolensis*	0	0	1	0	1	0.2	Native
*Piliostygma thonningii*	0	0	1	0	1	0.2	Native
*Pinus radiata*	0	0	0	1	1	0.2	Exotic
*Punica granatum*	0	0	1	0	1	0.2	Exotic
Total	101	82	202	145	530	100	13/19 N/E
Mean/Residential plot	4.6	3.7	9.2	6.6	6.0		

*Note:* A survey was conducted in 22 residential plots for each neighborhood (total = 88). The mean area of plots = 656.3 m^2^.

Abbreviations: GCM, Gécamines; N/E, native/exotic; PP, Penga Penga.

The species richness varied among the neighborhoods, with higher values where the soil trace metal concentrations are lower (Kalebuka and Kaleja: 23 and 22, respectively) compared to the soil with elevated metal contamination (Gécamines and Penga Penga: 12 and 18, respectively) (Figure 1).

## 4 Discussion

### 4.1 Expectations of the Residents on ES Provided by Trees

Overall, these findings reveal that residents in these four neighborhoods of Lubumbashi do recognize and appreciate the multiple benefits of trees in their environments and have made diligent efforts to plant trees. Trees are needed for the provisioning services (fruit, medicine), regulating services (shade, wind break, cooling air, dust barrier, preventing soil erosion), and cultural services (aesthetics).

Fruit supply by trees, followed by shade provision and windbreak effects, were the services mostly expected of urban residents. This shows the need for food in the residential plot (Shackleton et al. 2015) and the scarcity of trees in the area at the time of plot occupation, so trees are needed to meet these expectations. Apart from the fact that the Penga Penga area was void of trees because of the degradation caused by air and soil pollution from mineral processing (Leteinturier et al. 1999; Shutcha et al. 2018), Useni et al. (2018) also showed that the expansion of the building at Lubumbashi led to a reduction of natural habitats, which implies the loss of trees in a newly allotted area. However, residents of Kalebuka, Gécamines and Kaleja tended to expect specifically more fruits than those of Penga Penga. The fact that Penga Penga residents expected more regulating services (shade, windbreak and decreasing dust in the air) than provisioning (fruit and medicinal plants) might be due to the severity of soil pollution in this environment (Shutcha 2010; Shutcha et al. 2015; Shutcha et al. 2018) and then regulating services are a priority. The service expectations from trees show the nature of the living area (Shackleton et al. 2015). It is also worth noting that most of the trees found in the Gécamines neighborhood ( *M. indica* ) were planted before the pollution of soil and the coming of residents, which could have influenced the residents' perception of ES provided by trees in this neighborhood. The residents in all neighborhoods disclosed that they are already enjoying these services from trees that they planted and the fruits that are produced (mainly mango, avocado, guava, lemon and papaw) are consumed and sometimes sold. The lower rate of expected ecosystem services, such as air quality regulation and erosion prevention, provided by trees may be justified by their ignorance of other very useful benefits of trees in their environment.

### 4.2 Diversity of Tree Species in the Four Neighborhoods

The polluted neighborhoods of Penga Penga and Gécamines had a lower number of trees per neighborhood and residential plot, and a poor number of species compared to the unpolluted neighborhoods (Kalebuka and Kaleja). The lower taxonomic richness in the polluted areas explains what most of Penga Penga and Gécamines residents testified during surveys that there were several trials and failures in growing trees before getting a mature tree for those who succeeded, because of the soil's hostility to sustaining plant growth, while residents of Kalebuka and Kaleja did not report such experience. Some experiments conducted with tree species on tailing soils (at Kipushi) and the contaminated soils by metalliferous fallout (at Lubumbashi) testified to a good performance *A. auriculiformis* , *A. lebbeck, S* *. guineense* , *L. leucocephala, A* *. polyacantha* *and P. guajava* in the phytostabilisation (Mwanasomwe, Langunu, Shutcha, and Colinet 2022; Mwanasomwe, Langunu, Nkulu, et al. 2022; Langunu et al. 2025). However, *M. indica* showed poor survival and growth in the study of Langunu et al. (2025), probably because of the only one variety that was used as planted material. Other tree species observed at Penga Penga or Gécamines neighborhoods are to be checked and tested for selecting good candidate species for phytostabilization (Berti and Cunningham 2000; Mendez and Maier 2008; Dary et al. 2010) in urban and periurban areas in the Copperbelt region. The contamination status of species between the polluted and the unpolluted neighborhood was studied by Mwanasomwe (2022) showing that leaves, barks, wood and fruits from polluted neighborhood (Penga Penga) had slightly higher mean concentration in Cu, Co and Zn compared to the unpolluted neighborhood (Kalebuka) underlining that the use of fruiting species (*P. guajava, M. indica, and P. americana*) and non‐fruiting species (*A. auriculiformis, L. leucocephala, and S. guineese*) for phytostabilization of polluted soils in Lubumbashi (Penga Penga) would not pose a major risk of contamination of the food chain if regular consumption of the leaves is avoided and given that mangoes and avocados did not show any worrying metal concentrations, except guavas, whose cobalt concentration was slightly above the threshold in Penga Penga. Langunu et al. (2023) added that the metal concentrations were higher in the fruits and leaves from Penga Penga, with 47% of samples above the FAO and WHO thresholds (vs. 18.5% in Kalebuka). The exotic trees were the most planted in all four neighborhoods, ever since exotic tree species are mostly used in reforestation (Carpenter et al. 2004), and they represent a substantial component of urban trees in cities all over the world (Sjöman et al. 2016), which has been the case in Lubumbashi (Useni et al. 2022; Useni et al. 2024). The report from residents in all neighborhoods underlined that the native trees (miombo species) observed were preserved seedlings found at the time of plot occupation. The presence of trees on a plot was determined by the owner's efforts, soil contamination, and willingness to allow trees to occupy a part of their plot. However, the presence of a given species on the plot was determined by the availability of planting material, the benefits or services to be obtained, and the success in establishing a chosen species on the plot. The tree planting material consisted of seedlings (Penga Penga 85% and Kalebuka 50%), seeds (Penga Penga 30% and Kalebuka 50%), and cuttings (Penga Penga 0% and Kalebuka 3%), data not shown.

Native tree species were shown to be more dominant compared to exotic in Chennai metropolitan city in India (Muthulingam and Thangavel 2012), but this study concerned the urban forests, which should be considered differently from individual trees in residential plots (Roy et al. 2012; Selmi 2014). Moreover, thirty‐four species were recorded overall, and fruiting exotic species dominated, with *M. indica* (44.7%) and *P. americana* (21.2%) being the most abundant both in the polluted and the unpolluted neighborhoods, which confirms the fact that fruit supply was the service most expected by residents overall (Table 2). This shows that residents in these neighborhoods (suburbs) need trees to get food (verbal testimonies of the respondents), which is included in the list of activities related to urban agriculture. In developing countries, urban agriculture plays a key role in reducing food insecurity and urban poverty (Zezza and Tasciotti 2010). Shackleton et al. (2015) showed that residents living in informal settlements ranked food supply as the first reason for planting or preserving trees, while those in the townships enjoyed the splendor that such trees provide. The fruiting species, i.e., *M. indica* , *P. americana* , *E. guineensis* , *M. sinensis* , *C. lemon* and *P. guajava* had higher number of trees in the unpolluted neighborhoods (Kalebuka and Kaleja). In contrast, the exotic non‐fruiting species *A. auriculiformis* , as well as some miombo species, were more abundant at Penga Penga (Table 4), which reveals that trees in the metal‐rich areas are much more needed for regulating services than provisioning services, whereas provisioning services (fruits) are more needed than regulating services in the unpolluted neighborhoods (Shackleton et al. 2015). However, it is worth noting that the presence of *A. auriculiformis* was reinforced by the city council, which facilitated the distribution and planting of this fast‐growing species in some areas for urban and peri‐urban reforestation.

### 4.3 World's Most Reported Services of Tree Species Frequently Planted at Lubumbashi and Implications for Phytostabilisation

Trees within urban landscapes are found in both public and private spaces. Their benefits, values, and meanings for residents may vary with time (Mincey et al. 2013), the type of environment (Pradhan et al. 2020), and inhabitant standard of living (Shackleton et al. 2015); urban green spaces safeguard quality of life in urban areas by contributing provisions for livelihood like foodstuffs and medicines while enriching human well‐being through providing and sustaining a clean environment along with satisfying spiritual, aesthetic, and social needs of inhabitants (Nowak et al. 2008; McPherson et al. 2011). Phytostabilisation is the only suitable method for the rehabilitation of contaminated soils in the Katangan Copperbelt because of the elevated metal concentration and large size of polluted lands (Dubourguier et al. 2001); given their structure and characteristics, tree species present more advantages in the reclamation of contaminated lands compared to grasses (Pulford and Watson 2003).

**FIGURE 2:**
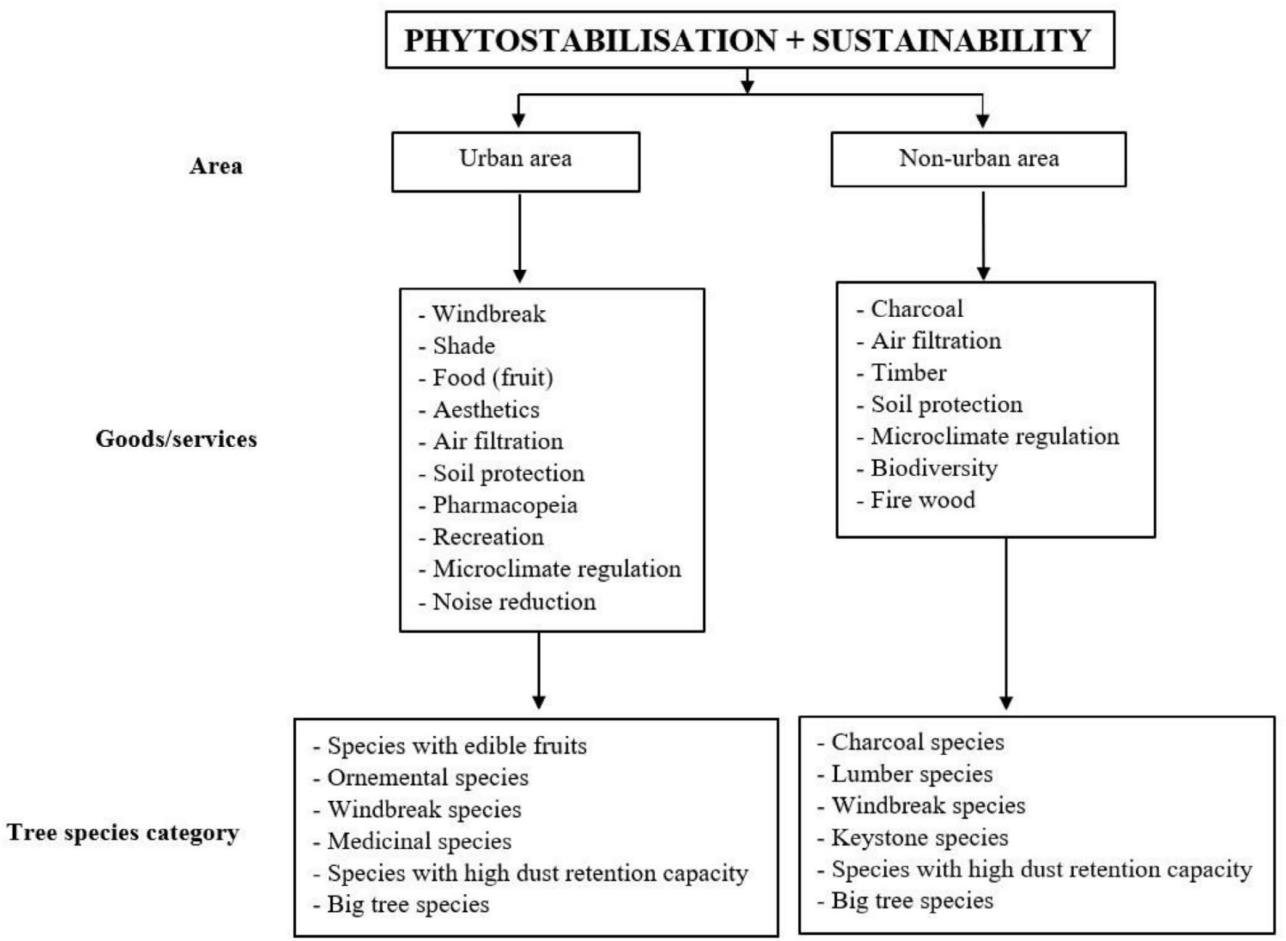
Decision process for phytostabilisation in urban and peri‐urban polluted areas.

The three steps of the decision process (Figure 2) lead to the combination of phytostabilisation with sustainable land use, offering the advantage of achieving environmental, economic, and social benefits while remediating contaminated soils in urban or non‐urban areas (Cundy et al. 2016). Figure 2 outlines the approach to be followed for the reclamation of trace metal‐polluted lands while obtaining suitable services according to the type of area (urban or peri‐urban), using tree species in the context of the Katangan Copperbelt. Step 1: type of contaminated area (urban or non‐urban area), step 2: categories of ES that are expected from the rehabilitation, step 3: choice of a species that can provide multiple services. Tree species that can provide multiple ES and respond well to the phytostabilisation criteria (such as high tolerance to metal contamination, rapid growth, high biomass, and lower translocation of metal from root to shoot) are a good option for sustainable phytostabilisation. Some of the tree species planted at Penga Penga and Gécamines might meet the residents' needs given their abundance (tolerance to metals) and the ES that they can provide (fruit, shade, windbreak, etc.), species such as *M. indica* , *P. americana* , *P. guajava* , *A. auriculiformis* , *S. guineense* , *B. spiciformis*. However, the concentration of metals in the edible fruits, leaves, and wood of these tree species should be well known before using them for both phytostabilisation and ES production, such as food, feed, charcoal, firewood, etc.

## 5 Conclusion

Destruction of the natural ecosystem and the environmental degradation of city quality due to rapid urbanization pose a dilemma when associated with pollution of soil by trace metals due to mining activity. This study sheds light on strategies to be adopted by policy planners to provide remedies to the environmental negative effects through the needs and behavior of residents in Lubumbashi (polluted and unpolluted) neighborhoods. Therefore, results showed that most trees that were grown by residents in the Lubumbashi neighborhoods perfectly revealed the kind of environment in which they live, e.g., the residents living in the polluted neighborhood (Penga Penga) desired more regulating services than those living in the unpolluted suburbs, while fruits (food) were the most desired overall. Moreover, the number of trees and species was higher in the unpolluted neighborhoods (Kalebuka and Kaleja) compared to the polluted neighborhoods (Penga Penga and Gécamines) and was characterized by a dominance of fruit tree species ( *M. indica* , *P. americana* , *C. papaya* and *P. guajava* ), whereas Penga Penga led with the non‐fruit species ( *A. auriculiformis* and *B. spiciformis*). These results provide guidelines on the choice of species according to their behavior toward polluted and unpolluted soils, and the reported ecosystem services on them in relation to the type of areas (urban or non‐urban areas) for sustainable reclamation in the Copperbelt region.

## Funding

This work was supported by Académie de recherche et d'enseignement supérieur.

## Conflicts of Interest

The authors declare no conflicts of interest.

## Supporting information


**Data S1:** pei370179‐sup‐0001‐DataS1.xlsx.

**Data S2:** pei370179‐sup‐0002‐DataS2.xlsx.

**Data S3:** pei370179‐sup‐0003‐DataS3.xlsx.

**Data S4:** pei370179‐sup‐0004‐DataS4.xlsx.

**Data S5:** pei370179‐sup‐0005‐DataS5.xlsx.

**Table S1:** pei370179‐sup‐0006‐TableS1.docx.

## Data Availability

The data used in this study are available and have been provided as Supporting Information for review. These data include: (i) data of soil metal concentration, (ii) the survey questionnaire, (iii) respondents' sociodemographic characteristics, (iv) survey responses regarding ecosystem services across four suburbs, (v) the number of trees recorded in residential plots within each suburb, and (vi) the diversity of tree species identified in the four suburbs of Lubumbashi.
