# Supplementary material for: Comparative Study of Expected Ecosystem Services and Tree Species Diversity in Residential Plots of Polluted and Unpolluted Neighborhoods in the Copper City of Lubumbashi
Source: Plant Environ Interact. 2026 Jul 14;7(4):e70179. doi: 10.1002/pei3.70179 (PMC13366389; doi:10.1002/pei3.70179)
Supplement: Supplementary file 6 — Table S1: pei370179‐sup‐0006‐TableS1.docx. [file PEI3-7-e70179-s003.docx]

**Table 1. Questionnaire items (questions and multiple-choice responses)**

1. Which is the year of your installation in the plot

2. The main reason for having trees in your homestead (expected services)

(a) aesthetics (b) protection against water erosion (c) protection against dust (d) getting fruits (e) wind break (g) (h) shade (i) medicinal plant (j) other

3. Total number of tree species and individuals in the residential plot

4. Gender of respondents

a. male b. female

5. Age

6. Educational level of the respondents

a. none b. undergraduate c. graduate d. primary e. secondary
